# Lumican is elevated in the lung in human and experimental acute respiratory distress syndrome and promotes early fibrotic responses to lung injury

**DOI:** 10.1186/s12967-022-03597-z

**Published:** 2022-09-04

**Authors:** Ke Wang, Youyu Wang, Yufang Cao, Hao Wang, Yongfang Zhou, Lijuan Gao, Zijian Zeng, Mengxin Cheng, Xiaodong Jin, Jun Chen, Fuqiang Wen, Tao Wang

**Affiliations:** 1 Division of Pulmonary Diseases, State Key Laboratory of Biotherapy, and Department of Respiratory and Critical Care Medicine, West China Hospital of Sichuan University, No. 37, Guoxue Alley, Chengdu, 610041 Sichuan China; 2 Department of Thoracic Surgery, Sichuan Academy of Medical Sciences & Sichuan Provincial People’s Hospital, Chengdu, China; 3 Department of Critical Care Medicine, Affiliated Haikou Hospital of Xiangya Medical College, Central South University, Haikou, China; 4 Department of Critical Care Medicine, West China Hospital of Sichuan University, Chengdu, China

**Keywords:** Acute respiratory distress syndrome, Early phase, Inflammation, Lumican, Lung injury, Profibrotic response

## Abstract

**Background:**

Fibroproliferative repair starts early in the inflammatory phase of acute respiratory distress syndrome (ARDS) and indicates a poor prognosis. Lumican, a small leucine-rich proteoglycan, is implicated in homeostasis and fibrogenesis, but its role in ARDS is unclear.

**Methods:**

Bronchoalveolar lavage fluid (BALF) samples were obtained from ARDS patients (n = 55) enrolled within 24 h of diagnosis and mechanically ventilated (n = 20) and spontaneously breathing (n = 29) control subjects. Lipopolysaccharide (LPS)-induced acute lung injury (ALI) mouse models were intratracheally administered an adeno-associated virus (AAV) vector expressing lumican shRNA. Primary human lung fibroblasts (HLF) and small airway epithelial cells (SAECs) were cultured with tumour necrosis factor (TNF)-α or lumican. Luminex/ELISA, histochemistry/immunohistochemistry, immunofluorescence microscopy, quantitative real-time PCR, and western blotting were performed.

**Results:**

Lumican levels were significantly higher in the BALF of ARDS patients than in that of ventilated or spontaneously breathing controls (both *p* < 0.0001); they were correlated with the PaO_2_/FiO_2_ ratio and levels of proinflammatory cytokines (interleukin-6, interleukin-8, and TNF-α) and profibrotic factors (fibronectin, alpha-1 type I collagen [COL1A1], and alpha-1 type III collagen [COL3A1]). Lumican expression was enhanced in the alveolar walls and airway epithelium in the ALI mouse model. Murine lumican levels were also linked to proinflammatory and profibrotic cytokine levels in the BALF. In vitro, TNF-α induced the synthesis and secretion of lumican in HLF. In turn, lumican increased the expression of alpha-smooth muscle actin (α-SMA), COL1A1, and COL3A1 in HLF, upregulated α-SMA and COL3A1, downregulated E-cadherin, and caused spindle-shaped morphological changes in SAECs. Moreover, increased ERK phosphorylation and Slug were noted in both HLF and SAECs treated with lumican. In vivo, AAV-mediated knockdown of lumican inhibited the pulmonary production of fibronectin and COL3A1 and alleviated lung fibrotic lesions in LPS-challenged mice.

**Conclusions:**

Pulmonary lumican levels were increased early in human and experimental ARDS and linked to disease severity and inflammatory fibrotic processes. Lumican triggers the transdifferentiation of lung fibroblasts into myofibroblasts and epithelial-mesenchymal transition in SAECs, possibly via the ERK/Slug pathway. Knockdown of pulmonary lumican attenuated extracellular matrix deposition in ALI mice. Overall, lumican promotes fibrotic responses in the early phase of ARDS, suggesting its potential as a therapeutic target.

**Supplementary Information:**

The online version contains supplementary material available at 10.1186/s12967-022-03597-z.

## Introduction

Acute respiratory distress syndrome (ARDS), may result from conditions such as pneumonia, sepsis, aspiration, and trauma, and is characterised by progressive respiratory distress and refractory hypoxemia [1]. Although an increasing number of mechanisms have been implicated in ARDS, this syndrome still results in significant morbidity and mortality [2]. ARDS involves multiple pathological changes with an acute/early exudative organising or proliferative phase and a late-resolving or fibrotic phase [3]. The early exudative phase is usually accompanied by activation of many inflammatory cells and mass production of proinflammatory cytokines. The proliferative phase generally manifests as synthesis of extracellular matrix (ECM), proliferation of myofibroblasts, and epithelial-mesenchymal transition (EMT) [4]. Interestingly, recent studies suggest that the phases of ARDS occur in a continuum rather than in sequence [5, 6]. Early fibroproliferative activity has been identified in the lungs of patients with ARDS on histological assessment (lung tissue biopsy and autopsy) [7, 8], high-resolution computed tomography (HRCT) at ARDS onset [9], and serial measurements of procollagen concentrations in the blood and bronchoalveolar lavage fluid (BALF) within a few days of ARDS diagnosis [10, 11]. Pulmonary fibroproliferation in early ARDS predicts increased mortality, a longer duration of mechanical ventilation, a higher frequency of ventilation-associated complications (barotrauma and ventilator-associated pneumonia), and an increased susceptibility to multiple organ failure [9–13]. Therefore, fibroproliferation occurs early in ARDS and indicates a poor prognosis, suggesting the need to uncover possible therapeutic targets and underlying mechanisms.

During the fibroproliferative response, the early inflammatory phase of ARDS is usually accompanied by deposition of many ECM components [5, 14]. The small leucine-rich repeat protein (SLRP) family is one of the extracellular supramolecular assemblies, which are essential for maintaining the balance of surrounding structures and participate in many cellular functions [15–17]. Lumican is one of the type II SLRPs, which contain polylactosamine or keratan sulphate chains in their tandem leucine-rich repeats [15, 18]. It has been reported that lumican is necessary for regulation of activity in some tumours, including gastric cancer, pancreatic ductal adenocarcinoma, and breast cancer [19–21]. Lumican also regulates migration of epithelial cells [22], proliferation and apoptosis of fibroblasts [23, 24], and organisation of collagen fibrils [24–26]. In addition, lumican was found to act with proinflammatory cytokines in regulation of inflammation in mouse cardiac fibroblasts [27]. Lumican-deficient mouse macrophages showed impaired innate immune functions in response to lipopolysaccharides (LPS) exposure [28]. Furthermore, lumican could modulate EMT in high-tidal-volume mechanical ventilation-induced lung injury in mice [29]. However, the potential role of lumican and its interactive function in pulmonary cells in the early phase of ARDS are unclear.

We hypothesized that lumican may be involved in the inflammatory response and repair of injury early in the course of ARDS. This study sought to determine whether pulmonary lumican levels were associated with lung inflammation, profibrotic responses, and disease severity in ARDS, and, if so, to elucidate the possible role of lumican in the processes of acute inflammation and fibroproliferative repair, using a combination of clinical and in vitro studies and an animal model of acute lung injury (ALI) to explore the underlying mechanisms.

## Materials and methods

### Study population

Fifty-five subjects who were admitted to the intensive care unit (ICU) in West China Hospital with a diagnosis of ARDS according to the Berlin definition [30] were enrolled in the study. All patients with ARDS received mechanical ventilation. Twenty mechanically ventilated ICU patients who did not meet the criteria for ARDS served as ventilated controls. Twenty-nine patients from the outpatient clinic who had been referred for fibrobronchoscopy and in whom no clinically significant pulmonary disease was found were enrolled as spontaneously breathing controls. Patients younger than 18 years of age and those who were pregnant were excluded. All patients were screened and enrolled between December 2017 and April 2021. The study protocol was designed in accordance with the principles of the Declaration of Helsinki and approved by the Clinical Ethics Committee of West China Hospital, Sichuan University (approval number 2017.195). Informed consent was obtained from all patients or their legal representatives.

### Acquisition of clinical information and samples

Demographic and anthropometric data (age, sex, height, and weight), main presenting complaints, medical history, and other clinical information were obtained by careful history-taking. BALF samples were collected according to the American Thoracic Society guideline [31]. The criteria for contraindication to fibrobronchoscopy were cardiopulmonary instability or severe haemorrhagic diathesis. Samples collection (including serum and BALF) was performed within 24 h of diagnosis of ARDS. The same information was collected for the control subjects. The PaO_2_/FiO_2_ ratio, blood lactate, and blood pH values were collected from the first blood gas sample drawn after intubation in the patients with ARDS.

### Antibodies and reagents

Rabbit anti-lumican (cat.ab168348) and E-cadherin (cat.ab40772) monoclonal antibodies were purchased from Abcam (Cambridge, MA, USA). Rabbit anti-Slug (cat.9585), β-actin (cat.4970), alpha-smooth muscle actin (α-SMA; cat.19245), p44/42 MAPK (ERK; cat.4695), and phospho-p44/42 MAPK (p-ERK; cat.4370) monoclonal antibodies were purchased from Cell Signaling Technology (Beverly, MA, USA). Rabbit anti-alpha-1 type III collagen (COL3A1; cat.A3795) polyclonal antibody was purchased from ABclonal Technology (Wuhan, China). Mouse anti-fibronectin monoclonal antibodies (cat.250073) was purchased from Zen Bioscience (Chengdu, China). Alexa Fluor® 488 goat anti-rabbit (cat.4412) and Alexa Fluor® 594 goat anti-mouse (cat.8890) secondary antibodies were purchased from Cell Signaling Technology. Recombinant human interleukin (IL)-6, IL-8, tumour necrosis factor (TNF)-α, and lumican proteins were purchased from R&D Systems (Minneapolis, MN, USA).

### Animal model

All experimental procedures performed in animals were in accordance with ARRIVE (Animal Research: Reporting of In Vivo Experiments) guidelines and approved by the Animal Ethics Committee at West China Hospital of Sichuan University. Male C57BL/6 mice aged 6–8 weeks were purchased from the Beijing Huafukang Bioscience Co. Inc (Beijing, China). Mice were housed at specific pathogen-free animal facilities in a room maintained at a suitable temperature and humidity on a 12-h light/12-h dark cycle. After administration of isoflurane inhalation anaesthetic, mice were treated intratracheally with LPS (*Escherichia coli* O111:B4, Sigma-Aldrich, St. Louis, Missouri, USA) dissolved in saline at a dose of 5 mg/kg in a total volume of 50 μL for each mouse or 50 μL of saline alone as a vehicle control, using a non-invasive intratracheal MicroSprayer™ (Penn-Century Inc., Wyndmoor, PA, USA) as previously described by us [32]. On days 1, 3, and 7 after LPS challenge, the mice were euthanised by intraperitoneal administration of pentobarbital sodium (40 mg/kg) and exsanguinated by cardiac puncture. The left main bronchus was ligated, and the right lung was lavaged three times with 0.5 mL of sterile ice-cold phosphate-buffered saline (PBS) supplemented with protease inhibitors, with a recovery rate of more than 90%. The right lung lobes were then dissected, snap frozen, and stored in liquid nitrogen for further analysis. The left lung was removed and fixed in 4% neutralized formaldehyde solution at 4 °C for 24 h, and then embedded in paraffin and sectioned at 5 μm. For frozen lung sections, the left lung was injected 200 μL embedding solution (the mixture of 100 μL 4% paraformaldehyde and 100 μL optimal cutting temperature compound), then fixed in 4% paraformaldehyde for 24 h at 4 °C, followed by dehydration in 30% sucrose solution at 4 °C for 48 h. Then, the lung was cryosectioned as frozen slices at 5 μm and stored at -20 °C.

### In vivo* adeno-associated virus-9-mediated lumican gene knockdown*

The adeno-associated virus-9 (AAV-9) vector was generated after cloning mouse lumican short hairpin RNA (shRNA) fragments into the adeno-associated virus vector GV628 (Shanghai Genechem Co., Ltd., Shanghai, China). The recombinant AAV carrying mouse lumican shRNA or scrambled shRNA was intratracheally administered to mice at a dose of 3.5 × 10^11^ V.G/mL in 50 μL of PBS per mouse. Vehicle control mice received an equal volume of PBS. After four weeks, mice were intratracheally administered LPS for ARDS modelling, and thereafter sacrificed 24 h after LPS challenge.

### Histochemistry, immunohistochemistry, and immunofluorescence analyses

Lung tissue paraffin sections were stained with haematoxylin–eosin and Masson’s trichrome. For immunohistochemical analysis, the tissue sections were deparaffinised in xylene, rehydrated in alcohol, and after washing and normal serum blocking, were incubated with primary antibodies including human anti-lumican antibody (1:100) for 1 h at room temperature. Next, they were incubated with anti-rabbit secondary antibodies and conjugated streptavidin–horseradish peroxidase. Each tissue section was then covered with freshly prepared diaminobenzidine chromogen for a few minutes to demonstrate optimum staining. Finally, all slides were counterstained by haematoxylin and mounted after dehydration with alcohol and acetone. For immunofluorescence analysis, the lung frozen slices were washed in PBS, blocked for 1 h at room temperature in 5% bovine serum albumin (BSA) and 0.3% Triton, and incubated overnight at 4 °C with rabbit anti-lumican antibody (1:100) and mouse anti-fibronectin antibody (1:50). Next, the sections were incubated with fluorescent-dye conjugated secondary antibody (1:500). Finally, the sections were sealed with mounting medium containing 4′,6-diamidino-2-phenylindole (DAPI; Abcam, Cambridge, MA, USA). Images were captured by an Eclipse E800 microscope (Nikon, Japan).

### Cell culture

The primary human lung fibroblasts (HLF) and primary human small airway epithelial cells (SAECs) were purchased from Lifeline Cell Technology (Oceanside, CA, USA). The SAECs were cultured in BronchiaLife Epithelial Basal Medium supplemented with a BronchiaLife Life Factors Kit, and the HLF was maintained in FibroLife Fibroblast Basal Medium with a FibroLife S2 Fibroblast Life Factors Kit (all from Lifeline Cell Technology). All cells were cultured at 37℃ in a 5% CO_2_ environment.

### Cell stimulation

Based on the results of a cell viability assay (data not shown) and previous studies [25], the HLF and SAECs were treated with 10 ng/mL IL-6, 10 ng/mL IL-8, and 20 ng/mL TNF-α for 24 h at 70%–80% confluence. The SAECs were also treated with 25 ng/mL and 50 ng/mL recombinant human lumican. The total RNA was collected at 48 h and the total protein and cell culture supernatants were collected at 72 h after lumican treatment. The HLF were treated with 50 ng/mL and 100 ng/mL recombinant human lumican for 48 h, after which mRNA was collected. The protein and cell culture supernatants were collected after 72 h of treatment. For all the in vitro studies, three independent experiments were conducted with triplicate wells per treatment in each experiment.

### Real-time reverse transcription polymerase chain reaction (RT-PCR)

Total RNA was extracted using the E.Z.N.A. Total RNA kit I (Omega Bio-tek Inc, Norcross, GA, USA). Next, mRNA was reverse transcribed and synthesised into complementary DNA using the PrimeScript™ RT Reagent Kit with a gDNA Eraser (RR047A, Takara, Japan) following the manufacturer’s protocol. Real-time RT-PCR was performed in triplicate using FastStart Essential DNA Green Master (Roche, Penzberg, Germany). Primer sequences are listed in Additional file 1: Table S1. All results are presented as fold differences normalised to GAPDH. The data were analysed by the comparative threshold cycle method defined as 2^−ΔΔCT^.

### Magnetic luminex assay

Blood and BALF samples obtained from all human study participants and mice were centrifuged for 10 min at 230 g and 4℃. The supernatants were then collected, centrifuged for 15 min at 3685 g and 4℃, and stored at -80℃ until further measurements were performed. Lumican, fibronectin, IL-6, IL-8, and TNF-α levels of human serum and BALF were measured using a Human Magnetic Luminex Assay (R&D Systems, Minneapolis, MN, USA) on a Bio-Plex 200 system (Bio-Rad, Hercules, CA, USA) following the manufacturers’ instructions.

### Enzyme-linked immunosorbent assay (ELISA)

The collected cell culture supernatants were centrifuged immediately at 250 g for 5 min. The clear and transparent liquid on top was saved and stored at -80 °C. The concentration of lumican in the cell culture supernatants and human BALF alpha-1 type I collagen (COL1A1) levels were measured using ELISA kit (R&D Systems) according to the manufacturer’s instructions. Lumican levels in mouse BALF were measured using a mouse lumican ELISA kit (Biovision, Milpitas, CA, USA). Human COL3A1 and mouse COL3A1, fibronectin, and TNF-α levels in BALF were measured using ELISA kits purchased from DLDevelop (Wuxi, China).

### Western blotting analysis

Cells were harvested and the total protein concentration was measured using a bicinchoninic acid assay (Thermo Scientific, Waltham, MA, USA). Samples were probed with primary antibodies against COL3A1 (1:500), α-SMA (1:10,000), E-cadherin (1:1000), ERK (1:1000), p-ERK (1:1000), Slug (1:1000), and β-actin (1:1000) and incubated overnight at 4 °C. Secondary antibodies (anti-rabbit IgG, Cell Signaling Technology) were then diluted in Tris-buffered saline with Tween (TBST; 1:5000) and incubated for 1 h at room temperature. Signals were detected by a Tanon-5200 chemiluminescence system (Tanon Science & Technology Co., Ltd., Shanghai, China).

### Statistical analysis

The unpaired* t*-test or Mann–Whitney test was used to compared two groups depending on the distribution of the data. Data for three or more groups were analysed by one-way analysis of variance followed by Tukey’s post hoc test or Kruskal–Wallis test depending on the distribution of the data. Two-way ANOVA test was used to compare the means among groups with two independent variables. The relationship between two factors was examined using Spearman's correlation analysis (clinical data) or Pearson's correlation analysis (in vivo data). The statistical analysis was performed using SPSS for Windows version 20 (IBM Corp., Armonk, NY, USA) or GraphPad Prism version 8.0 (GraphPad Software, La Jolla, CA, USA). All the statistical figures were created by GraphPad Prism 8.0. A *p*-value of < 0.05 was considered statistically significant.

## Results

### Disposition of study participants

A total of 55 patients with ARDS, 20 mechanically ventilated controls, and 29 spontaneously breathing controls were enrolled in the study. The ARDS group included 24 patients with infectious pneumonia, 11 with non-pulmonary sepsis, 7 postoperative cases, 6 trauma-related cases, and 7 with other types of ARDS. There was no significant between-group difference in body mass index, age, or sex. The general clinical characteristics of the study participants are shown in Table 1.

**Table 1:** Demographic and clinical characteristics of the study participants

	Spontaeous breathing controls (N = 29)	Ventilated controls (N = 20)	ARDS patients (N = 55)	*p* value
Age (years)	50 [40–57]	52.0 [41.3–68.5]	56 [41–77]	0.090
Sex ratio (male/female)	21/8	17/3	43/12	0.582
BMI (kg/m^2^)	24.5 [21.7–26.6]	24.0[19.7–25.7]	25.5[23.0–27.7]	0.056
SOFA on admission		7 [4.3–9]	11 [8–13]	< 0.0001
PH		7.42 [7.39–7.43]	7.38 [7.27–7.44]	0.035
PaCO_2_ (mmHg)		39.2 [36.1–44.0]	39.8 [31.3–44.7]	0.710
PaO_2_ (mmHg)		106.5 [76.1–141.3]	78.8 [66.0–98.0]	0.002
FiO_2_ (%)		40 [31.3–40.0]	80 [60–100]	< 0.001
Lactate (mmol/L)		1.6 [1.3–1.8]	2.7 [1.8–4.2]	0.007
Oxygen saturation		99.2 [97.7–100]	95 [91–98]	< 0.0001
PaO_2_/FiO_2_ (mmHg)		331.6 [196.0–358.9]	106.7 [80.0–140.0]	< 0.0001
PEEP (cmH_2_O)		5 [5–6.5]	5 [5, 6]	0.319
Tidal volume (ml/kg IBW)		3.53 [3.25–4.21]	6.81 [6.21–7.29]	< 0.0001
Mechanical ventilation [n (%)]		20 (100%)	55 (100%)	
Duration of MV (d)		8.5 [3–25.75]	6 [3–10]	0.317
Mortality at day 28 [n (%)]		4 (20%)	21 (38.2%)	
Cause of death [n (%)]				
Sepsis		0	10 (47.6%)	
Pulmonary dysfunction		1 (25%)	5 (23.8%)	
Neurologic dysfunction		3 (75%)	2 (9.5%)	
Cardiac dysfunction		0	2 (9.5%)	
Multiple organ failure		0	2 (9.5%)	
ICU length of stay (d)		10 [4–26]	7 [4–13]	0.223
Causes for ICU admission [n (%)]				
Infectious pneumonia		0	24 (43.6%)	
Extra-pulmonary sepsis		0	11 (20%)	
Post-operation		14 (70%)	7 (12.7%)	
Trauma		1 (5%)	6 (10.9%)	
Others		5 (25%)	7 (12.7%)	

The data are presented as median [interquartile range] or frequencies (%)

*P* values were calculated using the Mann–Whitney test or Kruskal–Wallis test

*p* < 0.05 was considered statistically significant

*ARDS* acute respiratory distress syndrome, *BMI* body mass index, *FiO*_*2*_ fraction of inspiration O_2_, *IBW* ideal body weight, *ICU* intensive care unit, *MV* mechanical ventilation, *PaO*_*2*_ partial pressure of O_2_, *PaO*_*2*_*/FiO*_*2*_ partial pressure of O_2_/fraction of inspiration O_2_, *PEEP* positive end-expiratory pressure, *SOFA* sequential organ failure assessment

### BALF lumican was increased in the ARDS group and positively correlated with profibrotic factors

**Fig. 1:**
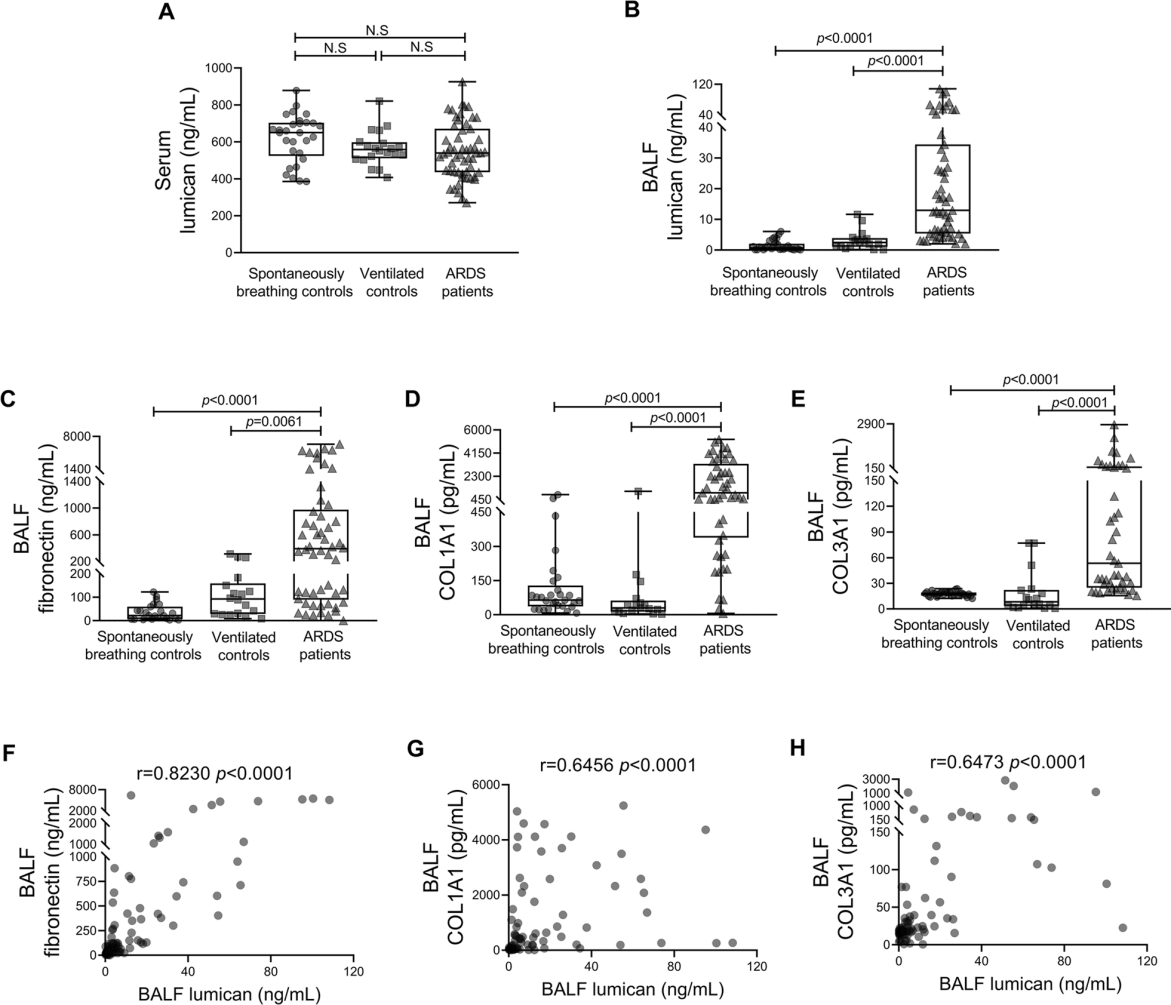
Lumican, fibronectin, COL1A1, and COL3A1 levels and their correlations in human subjects. Serum and BALF lumican levels (**A, B**) in the ARDS group and the control groups (spontaneously breathing controls and ventilated controls). BALF levels of fibronectin (**C**), COL1A1 (**D)**, and COL3A1 (**E**) were measured in all groups. BALF lumican levels were positively correlated with (**F**) BALF fibronectin levels, (**G**) BALF COL1A1 levels, and (**H**) BALF COL3A1 levels. Values are expressed as medians with interquartile ranges. The significance of between-group differences was examined using Kruskal–Wallis test. *p* < 0.05 was considered statistically significant. Correlations were investigated using Spearman’s correlation analysis (r indicates the correlation coefficient). *ARDS* acute respiratory distress syndrome, *BALF* bronchoalveolar lavage fluid, *COL1A1* alpha-1 type I collagen, *COL3A1* alpha-1 type III collagen

Although there was no significant difference in the serum lumican level between the ARDS group, the ventilated control group, and the spontaneously breathing control group (*p* > 0.05; Fig. 1A), the lumican level in BALF was significantly higher in the ARDS group (12,968.47 pg/mL [5387.91–34,446.62] pg/mL) than in the ventilated controls (2472.10 pg/mL [1070.86–3887.35 pg/mL]) and spontaneously breathing controls (677.87 pg/mL [291.27–2047.76 pg/mL]; *p* < 0.0001; Fig. 1B). Levels of fibronectin, COL1A1, and COL3A1 in BALF were also significantly higher in the ARDS group than in the control groups (all *p* < 0.0001; Fig. 1C–E). Furthermore, there was a positive correlation between the lumican level in BALF and the fibronectin, COL1A1, and COL3A1 levels in BALF in all subjects (Fig. 1F–H).

### *BALF lumican was correlated with BALF proinflammatory cytokine levels, the PaO*_*2*_*/FiO*_*2*_* ratio, and the SOFA score*

**Fig. 2:**
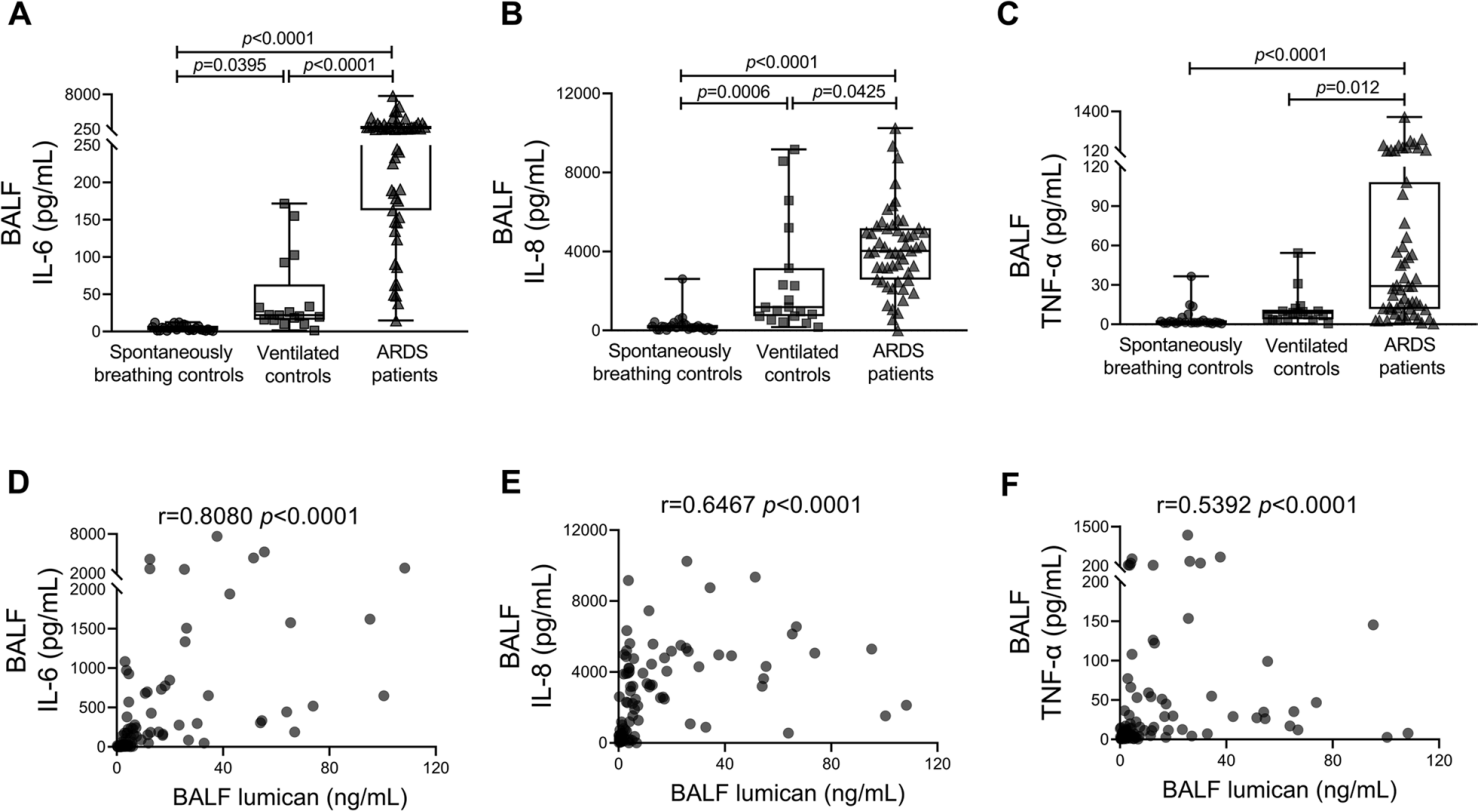
Proinflammatory cytokine levels and their correlation with lumican levels in human subjects. BALF levels of IL-6 (**A**), IL-8 (**B**), and TNF-α (**C**) were measured in patients with ARDS and control subjects. BALF lumican levels were positively correlated with BALF levels of (**D**) IL-6, (**E**) IL-8, and (**F**) TNF-α. Values are expressed as medians with interquartile ranges. The significance of between-group differences was examined using the Kruskal–Wallis test. *p* < 0.05 was considered statistically significant. Correlations were investigated using Spearman’s correlation analysis (r indicates the correlation coefficient). *ARDS* acute respiratory distress syndrome, *BALF* bronchoalveolar lavage fluid, *IL* interleukin, *TNF-α* tumour necrosis factor-alpha

Levels of the proinflammatory cytokines IL-6, IL-8, and TNF-α in BALF were considerably higher in the ARDS group than in the control groups (Fig. 2A–C). Spearman’s correlation analysis showed a positive correlation between the BALF lumican level and IL-6, IL-8, and TNF-α levels in BALF (Fig. 2D–F).

**Fig. 3:**
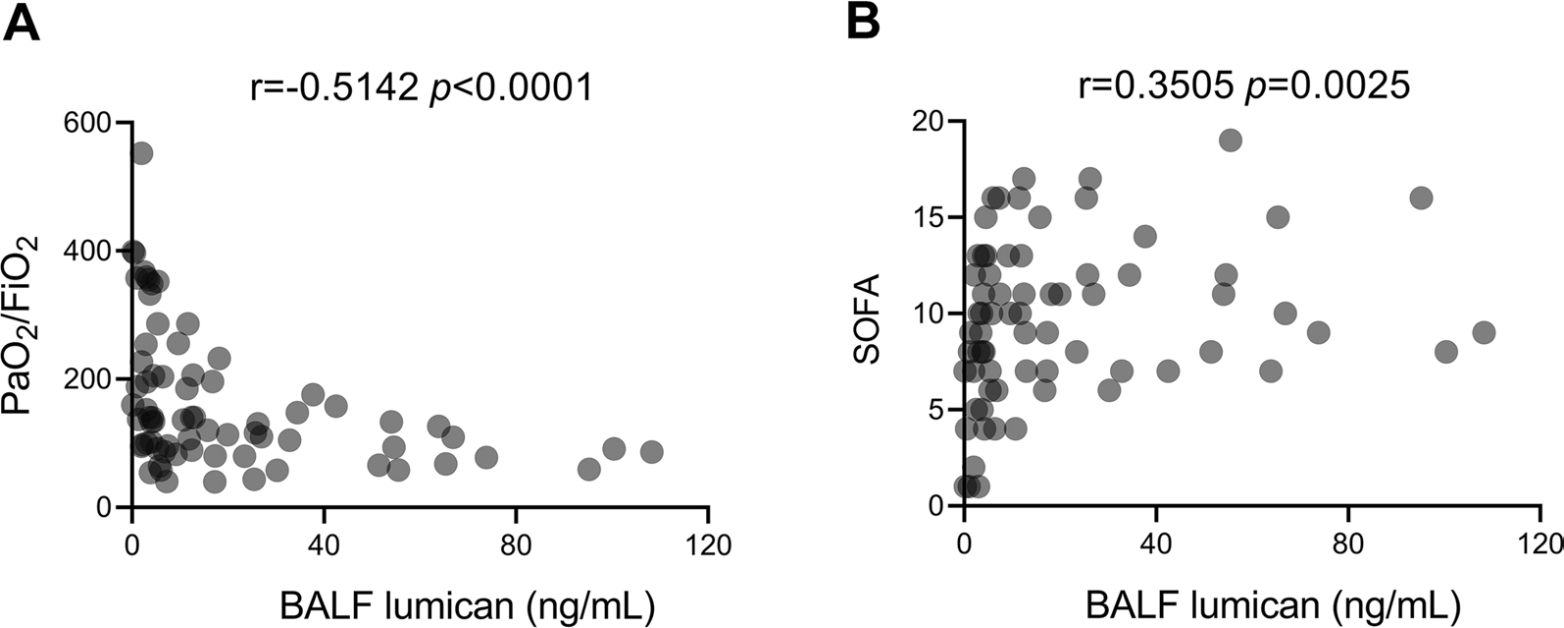
Relationships between the BALF lumican level, PaO_2_/FiO_2_ ratio, and SOFA score in human subjects. The BALF lumican level was negatively correlated with (**A**) the PaO_2_/FiO_2_ ratio and positively correlated with (**B**) the SOFA score. Correlations were investigated using Spearman’s correlation analysis (r indicates the correlation coefficient). *BALF* bronchoalveolar lavage fluid, *PaO*_*2*_*/FiO*_*2*_ partial pressure of oxygen/fraction of inspired oxygen, *SOFA* sequential organ failure assessment

We sought to identify the association between the BALF lumican level and the PaO_2_/FiO_2_ ratio, which defines acute hypoxic respiratory failure. We found a negative relationship between BALF lumican levels and the PaO_2_/FiO_2_ ratio (Fig. 3A; *r* = –0.5142, *p* < 0.0001). Moreover, the BALF lumican level was positively correlated with the SOFA (Sequential Organ Failure Assessment) score (Fig. 3B; *r* = 0.3505, *p* = 0.0025), which indicates the severity of critical illness in these patients.

### Lumican was increased in the lung of LPS-induced ALI mice

**Fig. 4:**
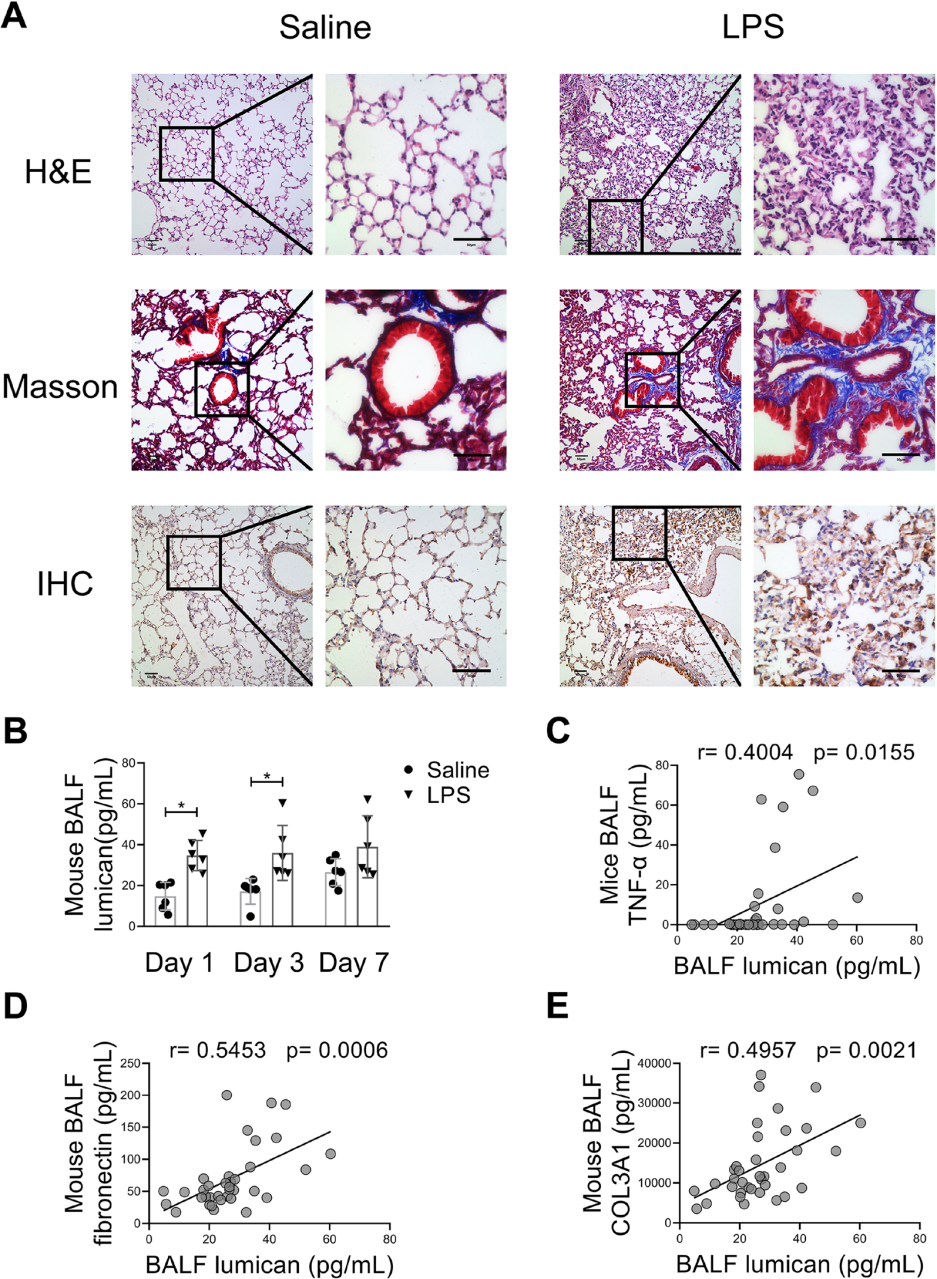
Pulmonary lumican expression was associated with inflammatory and profibrotic responses in an ALI mouse model. (**A**) Mice were intratracheally administered LPS (5 mg/kg) or saline as vehicle control. Lung tissue was collected 24 h after LPS challenge. Representative lung sections were stained with hematoxylin and eosin which labels cytoplasm and extracellular matrix in pink and nuclei in blue, Masson’s trichrome stain which labels collagen in blue, fibrin in red and cardiomyocytes in orange, and anti-lumican antibody which labels lumican in brown. Scale bars, 50 μm. (**B**) BALF lumican level was measured on days 1, 3, and 7 after LPS challenge. BALF lumican level was positively correlated with BALF levels of TNF-α (**C**), fibronectin **(D**), and COL3A1 (**E**). Each group consisted of a total of 6 mice. Values are expressed as the mean and standard deviation. The significance of between-group differences was examined using two-way analysis of variance followed by Tukey's multiple comparisons test to analyse the means of multiple samples. *p < 0.05 was considered statistically significant. Correlations were investigated using Pearson’s correlation analysis (r indicates the correlation coefficient). *BALF* bronchoalveolar lavage fluid, *COL3A1*, alpha-1 type III collagen, *LPS* lipopolysaccharides, *TNF-α* tumour necrosis factor-alpha

The lung tissue from LPS-challenged mice showed typical lung injuries, including diffuse alveolar damage, infiltration of inflammatory cells, and assembly of more fibres, as early as 24 h after LPS challenge on haematoxylin–eosin and Masson’s trichrome staining (Fig. 4A). In parallel, immunohistochemistry demonstrated increased expression of lumican in the alveolar walls and airway epithelium in mice with LPS-induced ALI (Fig. 4A). Furthermore, the fibrotic lesions in the lung remained unchanged on days 3 and 7 after LPS challenge (Additional file 1: Fig. S1 A).

### Correlation of BALF lumican with BALF proinflammatory and profibrotic cytokines in mice with LPS-induced ALI

The concentrations of lumican (Fig. 4B), TNF-α, fibronectin, and COL3A1 (Additional file 1: Fig. S1 B–D) in BALF were substantially higher in the ALI mice than in the control mice on day 1 after LPS challenge. It showed a trend of declining levels of BALF TNF-α and fibronectin from day 1 to day 7 while the lumican and COL3A1 levels remained relatively stable. Furthermore, there was a positive relationship between BALF lumican level and the TNF-α, fibronectin, and COL3A1 levels in BALF (Fig. 4C–E).

### TNF-α increased expression of lumican in HLF

**Fig. 5:**
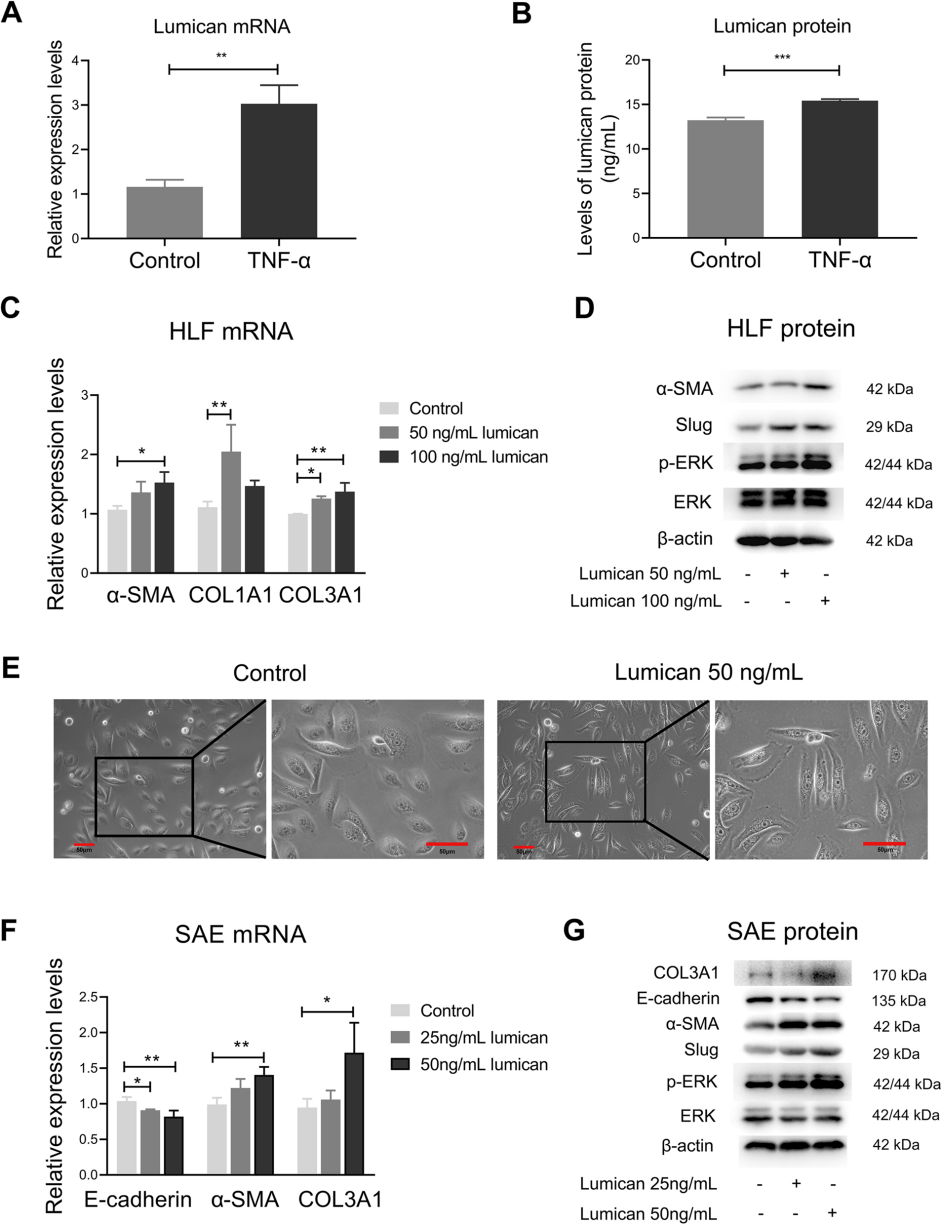
TNF-α increased lumican expression in HLF and lumican triggered fibroblast-to-myofibroblast transition and epithelial-mesenchymal transition. (**A**) Lumican mRNA levels were assessed by real-time reverse transcription polymerase chain reaction. (**B**) Lumican protein level was measured using enzyme-linked immunosorbent assay in cell culture supernatants. When lumican was incubated with HLF, expression levels of α-SMA, COL1A1, and COL3A1 mRNA were higher than those in control cells (**C**), as were the levels of α-SMA, Slug, and phosphorylated ERK protein (**D**). After culture with lumican for 24 h, SAECs changes to a spindle-like morphology (**E**). Scale bar, 50 μm. E-cadherin, α-SMA, and COL3A1 mRNA expression in lumican-treated SAECs are shown in (**F**). Representative protein levels in control cells and lumican-treated SAECs are shown as Western blots (**G**). Three independent experiments were conducted with triplicate wells per treatment in each experiment. The difference between two groups was examined using the unpaired *t*-test (n = 3). Values are expressed as the mean and standard deviation. The significance of between-group differences was examined using one-way analysis of variance followed by Tukey's multiple comparisons test to analyse the means of multiple samples. *p* < 0.05 was considered statistically significant. *p < 0.05, **p < 0.01, ***p < 0.005. *α-SMA* alpha-smooth muscle actin, *COL1A1* alpha-1 type I collagen, *COL3A1* alpha-1 type III collagen, *HLF* primary human lung fibroblasts, *SAECs* small airway epithelial cells, *TNF-α* tumour necrosis factor-alpha

TNF-α is a proinflammatory cytokine that has been reported to induce the release of lumican in cardiac fibroblasts [27]. Previous research showed that TNF-α is involved in development of ARDS and can modulate the fibrosis process in early ARDS [33]. In this study, to investigate the effects of TNF-α and lumican on activation of HLF, we stimulated the cells by administration of 20 ng/mL TNF-α (the concentration was chosen according to a previous study [25] and the cell viability test). After stimulation, we found a significant increase in expression of lumican mRNA (Fig. 5A). More lumican protein was secreted in the culture medium in the TNF-α-treated group than in the control group (Fig. 5B). However, neither IL-6 nor IL-8 had a significant effect on expression of lumican mRNA in HLF (Additional file 1: Fig. S2).

### Lumican regulated the fibroblast-to-myofibroblast transition in HLF

We next sought to explore the function of lumican released from HLF. We cultured HLF with different concentrations of recombinant lumican. Cells cultured with lumican showed a heightened expression of α-SMA mRNA, and similar results were obtained in a Western blotting assay (Fig. 5C, D). We also demonstrated that levels of phosphorylated ERK and Slug protein were higher in lumican-treated HLF than in control cells (Fig. 5D).

### Lumican regulated the epithelial-mesenchymal transition in SAECs

As observed by our own research team and other researchers [34], immunohistochemical staining revealed that lumican was also distributed in alveolar and small airway epithelial cells (Fig. 4A). Therefore, we treated SAECs with lumican (25 ng/mL and 50 ng/mL) to determine whether lumican has similar profibrotic effects in the epithelium of the lung. After culture with lumican for 24 h, SAECs showed a change to a spindle-like morphology (Fig. 5E), a decrease in the E-cadherin expression, and upregulation of both α-SMA and COL3A1 (Fig. 5F, G). Furthermore, phosphorylated ERK and Slug protein levels were elevated in lumican-treated SAECs (Fig. 5G).

### Lumican knockdown decreased pulmonary ECM deposition in mice with LPS-induced ALI

**Fig. 6:**
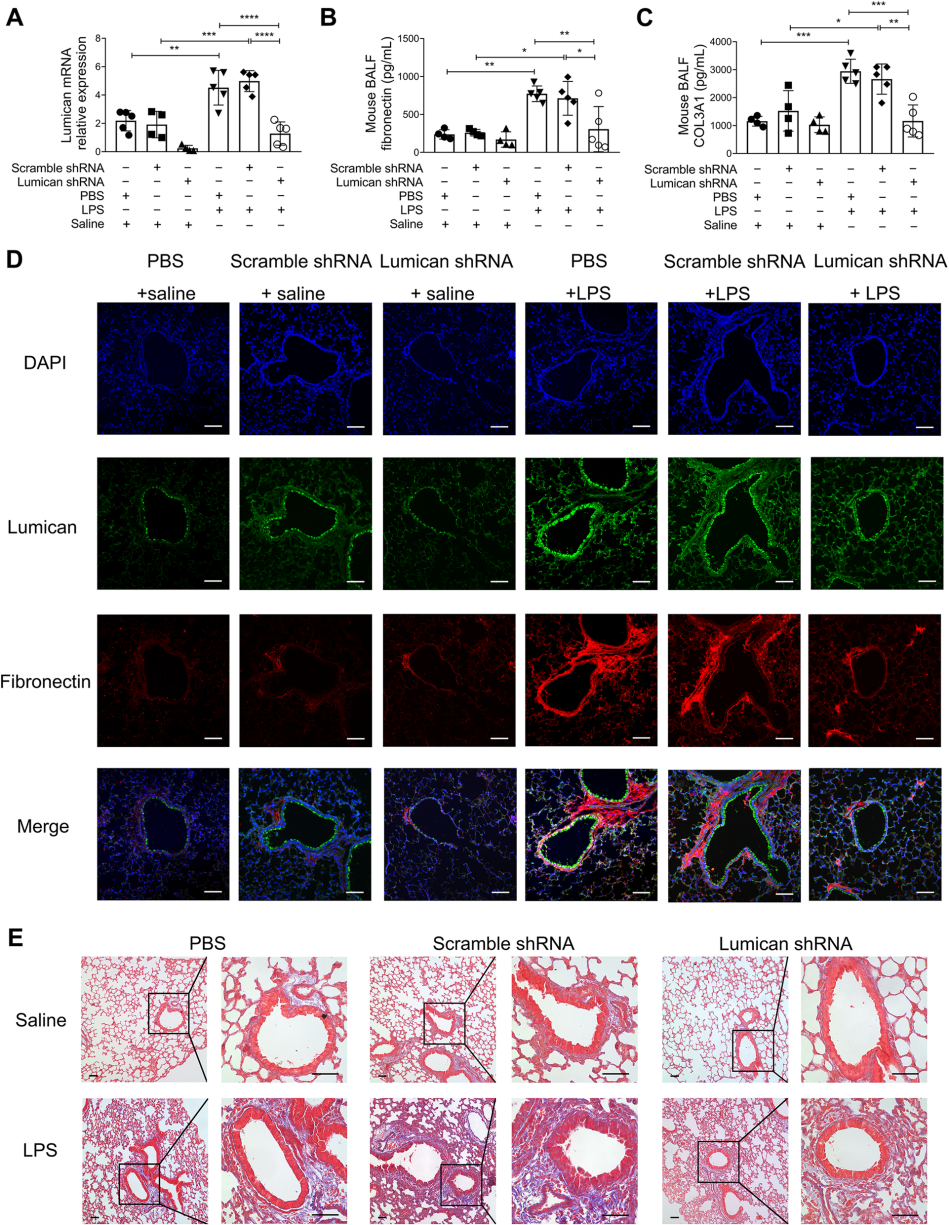
Lumican knockdown decreased ECM deposition in an LPS-induced ALI mouse model. Lumican mRNA levels in the lung tissue (**A**), and BALF levels of fibronectin (**B**) and COL3A1 (**C**) were assessed in mice intratracheally administrated with PBS or AAV-packed scrambled shRNA or lumican shRNA with or without LPS challenge at 24 h (n = 4–5 per group). Values are expressed as the mean and standard deviation. (**D**) Representative lung sections stained with immunofluorescence stain which labels DAPI in blue, lumican in green and fibronectin in red in mouse lung sections. Scale bar, 50 μm. (**E**) Representative lung sections stained with Masson’s trichrome stain in mice. The significance of between-group differences was examined using two-way analysis of variance followed by Tukey's multiple comparisons test to analyse the means of multiple samples. *p* < 0.05 was considered statistically significant. *p < 0.05, **p < 0.01, ***p < 0.005, ****p < 0.0001. *AAV* adeno-associated virus, *ALI* acute lung injury, *BALF* bronchoalveolar lavage fluid, *COL3A1* alpha-1 type III collagen, *DAPI* 2-(4-Amidinophenyl)-6-indolecarbamidine dihydrochloride, *ECM* extracellular matrix, *LPS* lipopolysaccharides, *PBS* phosphate-buffered saline, *TNF-α* tumour necrosis factor-alpha

To further confirm that lumican participates in pulmonary fibrotic process in the early phase of ALI in vivo, lumican knockdown in the lung was achieved through intratracheal administration of AAV-packed shRNA in mice. The results demonstrated that there was no difference in lumican expression between scrambled shRNA and PBS treatment, but lumican shRNA administration significantly decreased lumican expression in the lung tissue of both saline- and LPS-treated mice (Fig. 6A). Furthermore, the BALF levels of fibronectin and COL3A1 were significantly lower in lumican shRNA-treated mice than in scrambled shRNA-treated mice (Fig. 6B, C). Tissue immunofluorescence staining confirmed that AAV-mediated lumican shRNA delivery decreased lumican protein expression and inhibited LPS-induced fibronectin deposition in the lung (Fig. 6D). Moreover, Masson’s trichrome staining further confirmed that lumican knockdown alleviated fibrotic lesions in the early phase of lung inflammation in LPS-challenged mice (Fig. 6E).

## Discussion

There is mounting evidence that the classical three stages of ARDS, i.e., the initial inflammatory phase, fibroproliferation, and interstitial and intra-alveolar fibrosis, are not independent steps, but an inseparable and interactive process. For example, a previous study demonstrated potent mitogenic activity and increases in the levels of N-terminal procollagen peptide-III, two crucial mechanisms driving the deposition of lung collagen, in the BALF of patients with ARDS as early as 24 h after diagnosis [10]. A prospective cohort study of clinical autopsies in large population samples also revealed that fibroproliferative changes occurred early after the onset of ARDS; these changes were noted in more than half of patients with ARDS within the first week of evolution [7]. Moreover, fibroproliferative radiologic abnormalities were detectable on HRCT performed on the day of ARDS diagnosis, and higher HRCT scores predicted a poor prognosis and prolonged mechanical ventilation [9]. Similarly, in experimental ARDS animal models, a thin layer of type III collagen was found to be deposited in the alveolar septa of the mouse lung at 24 h after LPS challenge [35], and both collagen and elastic components were markedly increased in rats with paraquat-induced ALI as early as 24 h after induction of lesions [36]. In the present study, fibronectin, COL1A1, and COL3A1 levels were much higher in BALF samples collected from patients with ARDS within 24 h of diagnosis than in BALF samples collected from control subjects. Similar elevations of fibronectin were noted during the early inflammatory phase in a rat model of bleomycin-induced lung injury, which were accompanied by production of hyaluronan and development of pulmonary fibrosis [37]. Mice deficient in fibronectin containing extra type III domain failed to develop significant pulmonary fibrosis after bleomycin challenge [38]. It is well established that collagens, especially type I collagen, are strongly induced in the repair process, including lung fibrosis [39]. Accumulation of type III collagen, which forms fibrils and regulates their diameter, is also a crucial process in many chronic fibrotic diseases, including cardiac fibrosis and lung fibrosis [40]. A noteworthy finding of our study was that alveolar levels of lumican, an indispensable ECM component [17, 41], were markedly higher in the ARDS group than in the control (ventilated or spontaneously breathing) groups and were positively correlated with fibronectin, COL1A1, and COL3A1 levels. Furthermore, in our in vivo study, as expected, more lumican was deposited in the lung tissue of LPS-challenged mice than in that of saline-treated mice as early as 24 h after LPS challenge. Consistent with our findings in clinical subjects, the BALF lumican level was positively correlated with BALF fibronectin and COL3A1 levels in mice. These findings suggest that lumican released into the alveolar space is likely to play an important role in the early fibrotic responses in ARDS.

The acute onset of hypoxemia is a hallmark of ARDS, and refractory hypoxemia continues to pose a major treatment challenge and causes considerable mortality in ARDS [42, 43]. The PaO_2_/FiO_2_ ratio, measured by arterial blood gas analysis to assess the degree of hypoxemia, is crucial in the assessment of patients with ALI/ARDS [44]. This study provides new data indicating a positive relationship between the lumican level in BALF and the PaO_2_/FiO_2_ ratio. Moreover, we found a positive relationship between the BALF lumican level and the SOFA score. This scoring system is widely used for evaluation of severity of illness and predicting outcomes in patients with ARDS [45]. Furthermore, we observed that the levels of IL-6, IL-8, and TNF-α in BALF were much higher in the ARDS group than in the control group. It has been suggested that several proinflammatory cytokines are essential in the pathogenesis of ARDS. For example, IL-8 can trigger a neutrophil respiratory burst [46], TNF-α stimulates production of proinflammatory cytokines and increases oxidative stress [33], and when soluble IL-6 and its receptor anchor to membrane gp130, even stromal and epithelial cells can induce a marked inflammatory response in COVID-19 ARDS [47]. Interestingly, we found that lumican had a positive relationship with all of the above-mentioned proinflammatory cytokines in patients with ARDS and control subjects. In our mouse model of ALI, the levels of lumican, TNF-α, fibronectin, and COL3A1 in BALF were all increased as early as 24 h (day 1) after LPS challenge. Compared to the concentrations on day 1, TNF-α decreased markedly on days 3 and 7, while lumican and COL3A1 levels remained relatively stable over time. These results suggest that the fibrotic response is likely to start immediately after onset of injury and the inflammatory response and that the fibrotic process may continue for a period of time even when the inflammation has lessened.

Previous studies have demonstrated that lumican was often weakly expressed in peribronchial connective tissue, bronchial epithelium, and fibroblasts in normal lung tissue but was elevated in various pathological states [48, 49]. Our current study found that lumican expression was markedly increased in the lung tissue of LPS-challenged mice. Moreover, BALF lumican levels were significantly elevated in humans with ARDS and were positively correlated with the levels of IL-6, IL-8, and TNF-α in BALF. Therefore, we sought to determine whether these proinflammatory cytokines would stimulate constituent pulmonary cells to produce more lumican by stimulating HLF with IL-6, IL-8, and TNF-α. Interestingly, only TNF-α could activate HLF to produce more lumican. It has been suggested that TNF-α can stimulate secretion of lumican from fibroblasts to promote the differentiation of monocytes to fibrocytes [25]. Our present study findings suggest that increased release of lumican after lung injury may be attributed to increased release of proinflammatory cytokines early in the inflammatory course of ARDS.

Next, we sought to determine whether elevated lumican has its own biological activity in the injured lung in ARDS. As shown in a recent study, collagen cross-linking and myofibroblast transdifferentiation were significantly reduced after aortic banding in lumican-deficient mice [50]. Lumican was found to increase the expression of collagen in cardiac fibroblasts [27] and to have an important role in hepatic fibrosis [51]. Moreover, elevated lumican was shown to be involved in the process of EMT via the ERK pathway in a mouse model of ventilation-induced lung injury [29]. In order to determine the possible role of lumican in lung injury, we cultured primary human SAECs and HLF with different concentrations of recombinant lumican and found that lumican induced transformation of lung fibroblasts to myofibroblasts as well as EMT in SAECs. Accumulation of myofibroblasts is essential in pathological fibrosis and normal tissue wound healing [52]. In addition, when responding to injury, epithelial cells in the lung undergo EMT, which contributes to formation of fibrotic tissue [53]. Previous studies indicate that mutations in the ERK/ MAPK signalling pathway contribute to activation of the EMT program and progression of cancer [54]. Slug, a member of the Snail family of transcription factors, is reportedly related to EMT and its synthesis is modulated by ERK in wound healing [55]. In our study, we found that lumican significantly increased ERK phosphorylation and Slug expression in both HLF and SAECs. Thus, our findings indicate that lumican, which is induced by proinflammatory cytokines such as TNF-α, can induce both transdifferentiation of myofibroblasts to lung fibroblasts and EMT in pulmonary epithelial cells by activating the ERK/Slug signalling pathway. The function of lumican in pulmonary fibrotic processes in the early phase of lung injury was further explored in vivo. We demonstrated that AAV-mediated lumican knockdown significantly inhibited the production of ECM components including fibronectin and collagen and alleviated fibrotic lesions in the lung of ALI mice at 24 h after LPS challenge, confirming the potential role of lumican in the early profibrotic responses in ARDS development.

Our study has some limitations. First, although we found that lumican was upregulated in LPS-challenged mouse lung tissue, we could not obtain pulmonary histopathology in the early course of ARDS in our patients. Second, the changes in the fibrotic process and production of lumican in the lungs of patients with ARDS were not followed for long time after their diagnosis of ARDS. Nevertheless, to the best of our knowledge, this is the first study to demonstrate that BALF lumican levels are significantly increased in ARDS and show a positive correlation with clinical indices and levels of profibrotic and proinflammatory cytokines in BALF, implying a role of lumican after lung injury in the early course of ARDS. Furthermore, our combined in vitro and in vivo studies suggest that lumican production is induced upon inflammation and can promote fibrotic responses to lung injury.

## Conclusions

**Fig. 7:**
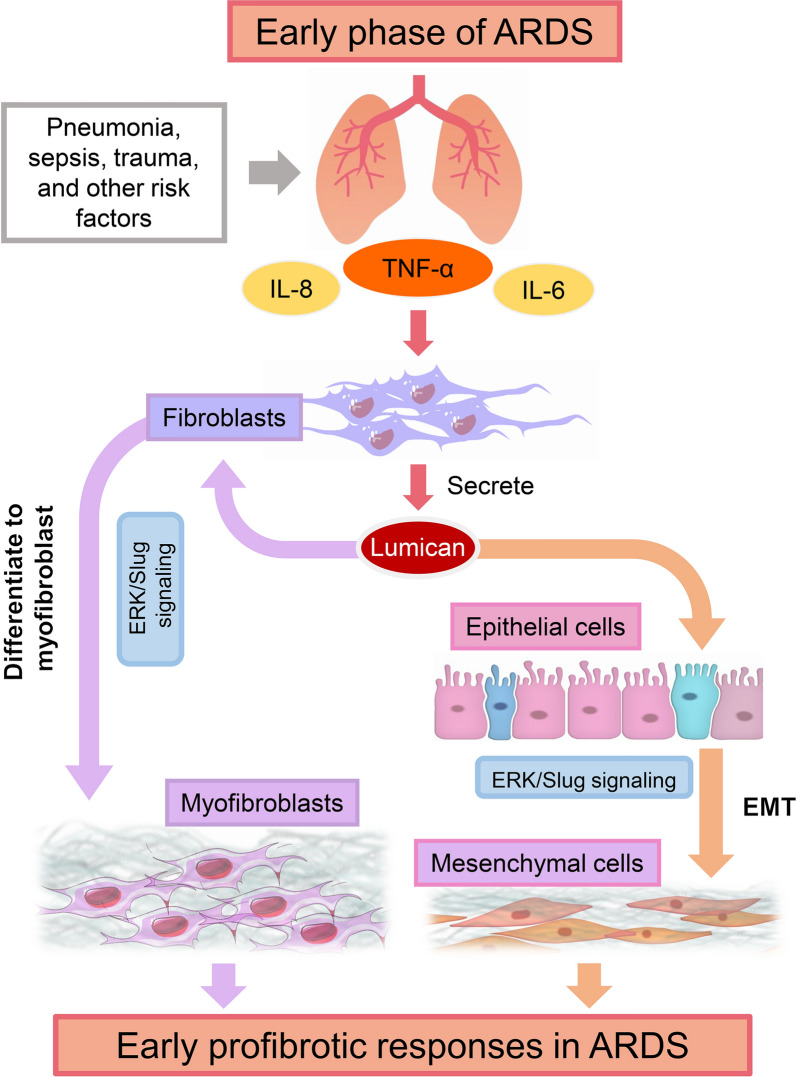
Schematic of the source and functional role of lumican in the early course of ARDS. TNF-α is a proinflammatory cytokine that is produced in the early phase of ARDS and induces the production of lumican in lung fibroblasts. Lumican then modulates fibroblast-to-myofibroblast transition and epithelial-to-mesenchymal transition (EMT) via the ERK/Slug signaling pathway, resulting in early profibrotic responses in ARDS. *ALI* acute lung injury, *ARDS* acute respiratory distress syndrome, *EMT* epithelial-to-mesenchymal transition, *IL* interleukin, *TNF-α* tumour necrosis factor-alpha

In summary, this study demonstrates that pulmonary levels of lumican are increased in the early phase of ALI/ARDS and are associated with lung inflammation, profibrotic responses, and disease severity. Further, the proinflammatory cytokine TNF-α induces lumican production from HLF and lumican can trigger transdifferentiation of myofibroblasts in HLF and EMT in SAECs via the ERK/Slug signalling pathway, thereby promoting fibrotic processes (Fig. 7). Furthermore, lumican knockdown in the lung tissue attenuates pulmonary ECM deposition and fibrotic lesions in LPS-induced ALI mouse model. Therefore, lumican may serve as a bridge between inflammation and fibrosis in the development of ALI/ARDS and may represent a novel therapeutic target for ARDS.

## Supplementary Information

**Additional file 1: Table S1.** qRT-PCR primer sequences. **Figure S1.** Histopathology and BALF levels of proinflammatory and profibrotic cytokines in mice after LPS challenge. (**A**) Representative images of lung sections stained with Masson's trichrome stain in mice on days 1, 3, and 7 after LPS challenge. Scale bars, 50 μm. The levels of TNF-α (**B**), fibronectin **(C**), and COL3A1 (**D**) in BALF were measured by ELISA on days 1, 3, and 7 after LPS challenge. Each group contained 6 mice. Values are expressed as the mean and standard deviation. The significance of between-group differences was examined using two-way analysis of variance followed by Tukey's multiple comparisons test to analyse the means of multiple samples. *p*<0.05 was considered statistically significant. *p<0.05, **p<0.01, ***p<0.005, ****p<0.0001. **Figure S2.** Lumican mRNA levels in primary HLF treated with IL-6 or IL-8. The lumican mRNA levels in HLF treated with or without 10 ng/ml IL-6 or 10 ng/ml IL-8 for 48 h were detected by real-time RT-PCR. Values are expressed as the mean and standard deviation. Three independent experiments were conducted with triplicate wells per treatment in each experiment (n=3). The significance of between-group differences was examined using analysis of variance followed by Tukey's multiple comparisons test to analyse the means of multiple samples. *p<*0.05 was considered statistically significant.

## Data Availability

The data used to support the findings of this study are available from the corresponding authors upon reasonable request.

## Abbreviations

AAV: Adeno-associated virus
ARDS: Acute respiratory distress syndrome
α-SMA: α-Smooth muscle actin
BALF: Bronchoalveolar lavage fluid
COL1A1: Alpha-1 collagen type I
COL3A1: Alpha-1 collagen type III
ECM: Extracellular matrix
EMT: Epithelial-to-mesenchymal transition
HLF: Human lung fibroblasts
LPS: Lipopolysaccharides
IL-6: Interleukin 6
IL-8: Interleukin 8
PaO_2_/FiO_2_: Partial pressure of O_2_/fraction of inspiration O_2_
PBS: Phosphate-buffered saline
SAECs: Human small airway epithelial cells
shRNA: Short hairpin RNA
SLRP: Small leucine-rich repeat protein
SOFA: Sequential organ failure assessment
TNF-α: Tumour necrosis factor-α
