# Supplementary material for: Lumican is elevated in the lung in human and experimental acute respiratory distress syndrome and promotes early fibrotic responses to lung injury
Source: J Transl Med. 2022 Sep 4;20:392. doi: 10.1186/s12967-022-03597-z (PMC9440861; doi:10.1186/s12967-022-03597-z)
Supplement: Supplementary file 1 — Additional file 1: Table S1. qRT-PCR primer sequences. Figure S1. Histopathology and BALF levels of proinflammatory and profibrotic cytokines in mice after LPS challenge. (A) Representative images of lung sections stained with Masson's trichrome stain in mice on days 1, 3, and 7 after LPS challenge. Scale bars, 50 μm. The levels of TNF-α (B), fibronectin (C), and COL3A1 (D) in BALF were measured by ELISA on days 1, 3, and 7 after LPS challenge. Each group contained 6 mice. Values are expressed as the mean and standard deviation. The significance of between-group differences was examined using two-way analysis of variance followed by Tukey's multiple comparisons test to analyse the means of multiple samples. p<0.05 was considered statistically significant. *p<0.05, **p<0.01, ***p<0.005, ****p<0.0001. Figure S2. Lumican mRNA levels in primary HLF treated with IL-6 or IL-8. The lumican mRNA levels in HLF treated with or without 10 ng/ml IL-6 or 10 ng/ml IL-8 for 48 h were detected by real-time RT-PCR. Values are expressed as the mean and standard deviation. Three independent experiments were conducted with triplicate wells per treatment in each experiment (n=3). The significance of between-group differences was examined using analysis of variance followed by Tukey's multiple comparisons test to analyse the means of multiple samples. p<0.05 was considered statistically significant. [file 12967_2022_3597_MOESM1_ESM.pdf]

Table S1 qRT-PCR primer sequences

Gene	Primer	Sequence	Product size (bp)	Organism
GAPDH	F	GGTGGTCTCCTCTGACTTCAACA	213	Homo
	R	TCTCTTCCTCTTGTGCTCTTGCT		sapiens
Gapdh	F	CATCACTGCCACCCAGAAGACTG	153	Mus
	R	ATGCCAGTGAGCTTCCCGTTCAG		musculus
LUMICAN	F	TCATCCATCTCCAGCACAATCG	250	Homo
	R	ATTCCACTATCAGCCAGTTCGTT		sapiens
Lumican	F	CCTCTCACACCGAACTGCCTCAT	216	Mus
	R	TCACGCCAGCCTCTGGATGAAGT		musculus
COL1A1	F	GATTCCCTGGACCTAAAGGTGC	107	Homo
	R	AGCCTCTCCATCTTTGCCAGCA		sapiens
COL3A1	F	GCTACGGCAATCCTGAACTTCC	236	Homo
	R	GCAACCATCCTCCAGAACTGTG		sapiens
α-SMA	F	TTACTACTGCTGAGCGTGAGATT	201	Homo
	R	CGATGAAGGATGGCTGGAACA		sapiens
E-cadherin	F	TACGCCTGGGACTCCACCTA	101	Homo
	R	CCAGAAACGGAGGCCTGAT		sapiens

Abbreviations: α-SMA, alpha-smooth muscle actin; COL1A1, alpha-1 type I collagen; COL3A1, alpha-1 type III collagen; GAPDH, Glyceraldehyde-3-Phosphate Dehydrogenase

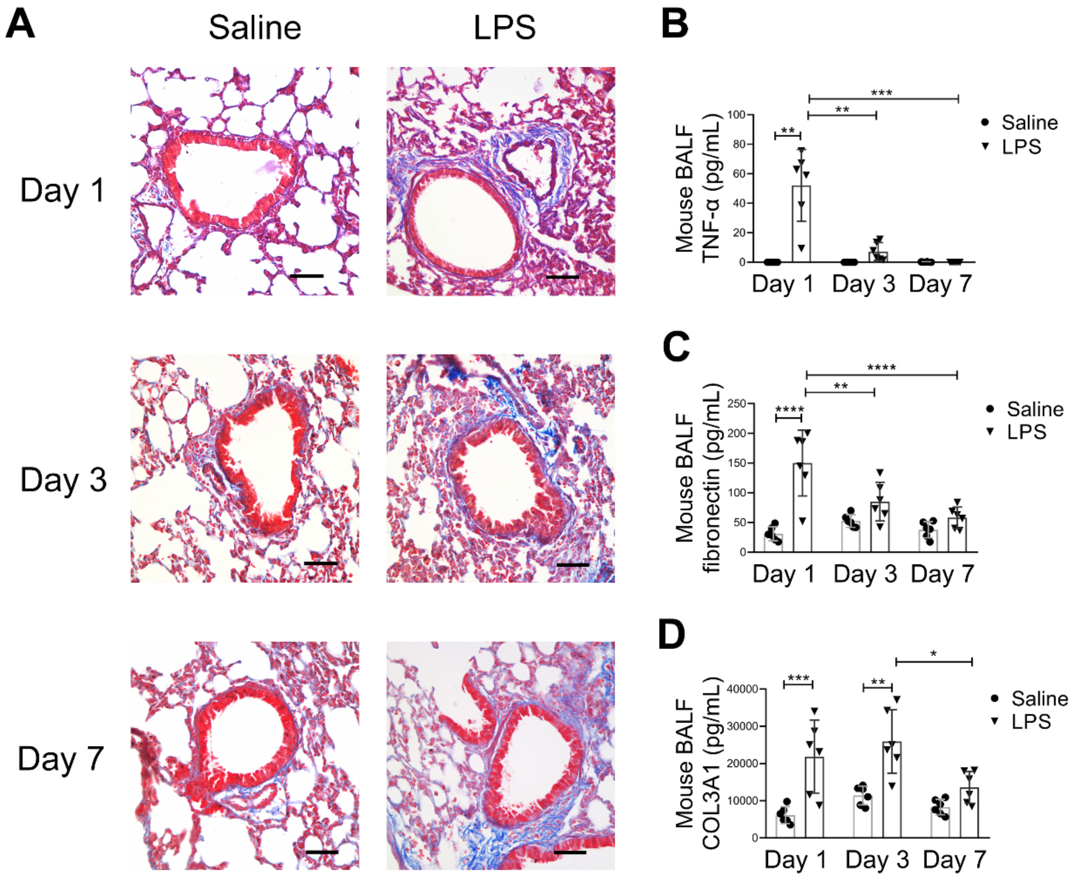

Fig. S1 Histopathology and BALF levels of proinflammatory and profibrotic cytokines in mice after LPS challenge. (**A**) Representative images of lung sections stained with Masson's trichrome stain in mice on days 1, 3, and 7 after LPS challenge. Scale bars, 50 µm. The levels of TNF-α (**B**), fibronectin (**C**), and COL3A1 (**D**) in BALF were measured by ELISA on days 1, 3, and 7 after LPS challenge. Each group contained 6 mice. Values are expressed as the mean and standard deviation. The significance of between-group differences was examined using two-way analysis of variance followed by Tukey's multiple comparisons test to analyse the means of multiple samples. $p<0.05$ was considered statistically significant. *$p<0.05$, **$p<0.01$, ***$p<0.005$, ****$p<0.0001$.

Abbreviations: BALF, bronchoalveolar lavage fluid; COL3A1, alpha-1 type III collagen; ELISA, enzyme-linked immunosorbent assay; LPS, lipopolysaccharides; TNF-α, tumor necrosis factor-alpha

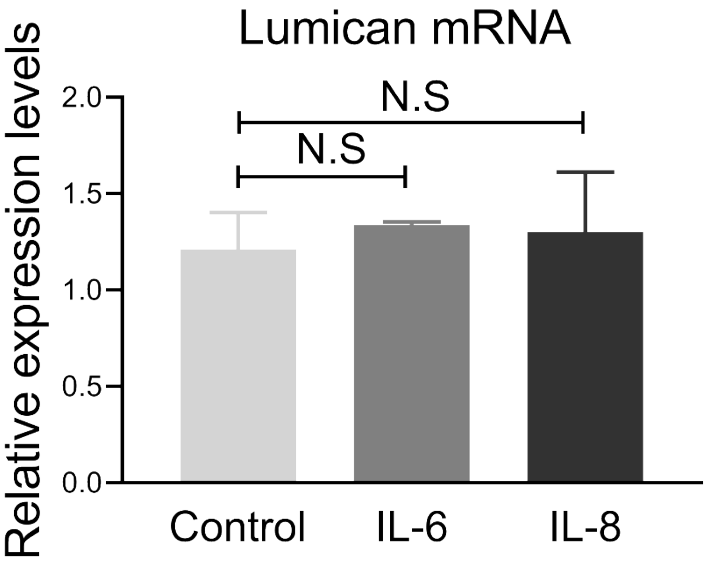

Fig. S2 Lumican mRNA levels in primary HLF treated with IL-6 or IL-8. The lumican mRNA levels in HLF treated with or without 10 ng/ml IL-6 or 10 ng/ml IL-8 for 48 h were detected by real-time RT-PCR. Values are expressed as the mean and standard deviation. Three independent experiments were conducted with triplicate wells per treatment in each experiment (n=3). The significance of between-group differences was examined using analysis of variance followed by Tukey's multiple comparisons test to analyse the means of multiple samples. $p<0.05$ was considered statistically significant.

Abbreviations: HLF, primary human lung fibroblasts; IL, interleukin; RT-PCR, reverse-transcription polymerase chain reaction
